# Supplementary material for: Evaluation of the diagnostic performance of EpiTuub® Fecal Rotavirus Antigen Rapid Test Kit in Amhara National Regional State, Ethiopia: A multi-center cross-sectional study
Source: PLoS One. 2023 Nov 30;18(11):e0295170. doi: 10.1371/journal.pone.0295170 (PMC10688889; doi:10.1371/journal.pone.0295170)
Supplement: S1 Checklist — (DOCX) [file pone.0295170.s001.docx]

STROBE Statement—checklist of items that should be included in reports of observational studies

|  | Item No. | Recommendation | Page  No. | Relevant text from manuscript |
| --- | --- | --- | --- | --- |
| Title and abstract | 1 | (a) Indicate the study’s design with a commonly used term in the title or the abstract | 1 | Cross-sectional study |
|  |  | (b) Provide in the abstract an informative and balanced summary of what was done and what was found | 2 | Sensitivity of 75.5% and specificity of 98.2% |
| Introduction | | | |  |
| Background/rationale | 2 | Explain the scientific background and rationale for the investigation being reported | 3-4 | No single diagnostic tool has been evaluated and approved for use in Ethiopian health care settings |
| Objectives | 3 | State specific objectives, including any prespecified hypotheses | 5 | To evaluate the diagnostic performance of the EpiTuub® Fecal Rotavirus Antigen Rapid Test Kit |
| Methods | | | |  |
| Study design | 4 | Present key elements of study design early in the paper | 5 | A multi-center hospital based cross-sectional study design |
| Setting | 5 | Describe the setting, locations, and relevant dates, including periods of recruitment, exposure, follow-up, and data collection | 5 | Three referral hospitals in Amhara National Regional state, Ethiopia between February 1, 2021 and December 31, 2022. |
| Participants | 6 | (a) Cohort study—Give the eligibility criteria, and the sources and methods of selection of participants. Describe methods of follow-up  Case-control study—Give the eligibility criteria, and the sources and methods of case ascertainment and control selection. Give the rationale for the choice of cases and controls    Cross-sectional study—Give the eligibility criteria, and the sources and methods of selection of participants | 5 | Under-five children with diarrhea visiting the hospitals |
|  |  | (b) Cohort study—For matched studies, give matching criteria and number of exposed and unexposed  Case-control study—For matched studies, give matching criteria and the number of controls per case |  |  |
| Variables | 7 | Clearly define all outcomes, exposures, predictors, potential confounders, and effect modifiers. Give diagnostic criteria, if applicable | 6-7 | The presence of red line in both test and control areas is interpreted as positive; the presence of red line only in the control area is interpreted as negative; and the absence of red line in the control area regardless of the test area is considered as invalid test result. A specific amplification with cycle threshold (CT) value of less than 40 was considered positive for rotavirus A infection. |
| Data sources/ measurement | 8* | For each variable of interest, give sources of data and details of methods of assessment (measurement). Describe comparability of assessment methods if there is more than one group | 5-6 | All samples stored at -80oC were tested for the presence of Rotavirus A antigen |
| Bias | 9 | Describe any efforts to address potential sources of bias |  | N/A |
| Study size | 10 | Explain how the study size was arrived at | 5 | Considering single population proportion formula, a total of 537 stool samples were collected |

Continued on next page

| Quantitative variables | 11 | Explain how quantitative variables were handled in the analyses. If applicable, describe which groupings were chosen and why |  |  |
| --- | --- | --- | --- | --- |
| Statistical methods | 12 | (a) Describe all statistical methods, including those used to control for confounding | 7 | The diagnostic performance of the EpiTuub® Fecal Rotavirus Antigen Rapid Test Kit was evaluated against the one-step RT-PCR. The sensitivity, specificity, positive and negative predictive values, and overall test accuracy of the rapid test kit with a 95% confidence interval was calculated |
|  |  | (b) Describe any methods used to examine subgroups and interactions |  | N/A |
|  |  | (c) Explain how missing data were addressed |  | N/A |
|  |  | (d) Cohort study—If applicable, explain how loss to follow-up was addressed  Case-control study—If applicable, explain how matching of cases and controls was addressed  Cross-sectional study—If applicable, describe analytical methods taking account of sampling strategy | 7 | The diagnostic performance of the EpiTuub® Fecal Rotavirus Antigen Rapid Test Kit was evaluated against the one-step RT-PCR. The sensitivity, specificity, positive and negative predictive values, and overall test accuracy of the rapid test kit with a 95% confidence interval was calculated. |
|  |  | (e) Describe any sensitivity analyses |  | NA |
| Results | | | | |
| Participants | 13* | (a) Report numbers of individuals at each stage of study—eg numbers potentially eligible, examined for eligibility, confirmed eligible, included in the study, completing follow-up, and analysed | 8 | A total of 537 children with diarrhea were included in the study. |
|  |  | (b) Give reasons for non-participation at each stage |  | N/A |
|  |  | (c) Consider use of a flow diagram |  | N/A |
| Descriptive data | 14* | (a) Give characteristics of study participants (eg demographic, clinical, social) and information on exposures and potential confounders | 8 | The mean age of the study participants was 25.5+15.2 months. The majority (97.6%) of the study participants were immunized against rotavirus. |
|  |  | (b) Indicate number of participants with missing data for each variable of interest |  | N/A |
|  |  | (c) Cohort study—Summarise follow-up time (eg, average and total amount) |  |  |
| Outcome data | 15* | Cohort study—Report numbers of outcome events or summary measures over time |  |  |
|  |  | Case-control study—Report numbers in each exposure category, or summary measures of exposure |  |  |
|  |  | Cross-sectional study—Report numbers of outcome events or summary measures | 9 | Among the study participants, 79 (14.71%) and 94 (17.5%) were positive for rotavirus with the rapid antigen test and One-step RT-PCR, respectively |
| Main results | 16 | (a) Give unadjusted estimates and, if applicable, confounder-adjusted estimates and their precision (eg, 95% confidence interval). Make clear which confounders were adjusted for and why they were included | 9 | The rapid fecal rotavirus antigen test kit is 75.53% (95% CI= 71.9-79.2) sensitive, 98.19% (95% CI=97.1-99.3) specific with a positive and negative predictive value of 89.9% (95% CI= 87.3-92.4) and 95% (95% CI=93.1-96.3), respectively |
|  |  | (b) Report category boundaries when continuous variables were categorized |  | N/A |
|  |  | (c) If relevant, consider translating estimates of relative risk into absolute risk for a meaningful time period |  | N/A |

Continued on next page

| Other analyses | 17 | Report other analyses done—eg analyses of subgroups and interactions, and sensitivity analyses | 10 | The overall accuracy of the rapid rotavirus antigen test kit is excellent with the area under the ROC curve of 86.9% (95% CI=81.6, 92.1%) |
| --- | --- | --- | --- | --- |
| Discussion | | | | |
| Key results | 18 | Summarise key results with reference to study objectives | 11 | In our study, the rapid fecal antigen test kit was found to have a sensitivity of 75.5% and a specificity of 98.2% with 23/458 (5%) false-negative and 8/79(10.12%) false-positive results |
| Limitations | 19 | Discuss limitations of the study, taking into account sources of potential bias or imprecision. Discuss both direction and magnitude of any potential bias | 13 | The study used only one kit for evaluation |
| Interpretation | 20 | Give a cautious overall interpretation of results considering objectives, limitations, multiplicity of analyses, results from similar studies, and other relevant evidence | 14 | The EpiTuub® Fecal Rotavirus Antigen Rapid Test Kit (KTR-917, Epitope Diagnostics, Sandiago USA) is a sensitive, specific, user-friendly, rapid, and equipment-free option to be used as a point of care test in Ethiopian healthcare settings where resource is limited to do one-step RT-PCR. |
| Generalisability | 21 | Discuss the generalisability (external validity) of the study results | 14 | Furthermore, the kit could be used in the evaluation and monitoring of rotavirus vaccine effectiveness in such settings. |
| Other information | |  | | |
| Funding | 22 | Give the source of funding and the role of the funders for the present study and, if applicable, for the original study on which the present article is based | 14 | This work was supported by the University of Gondar internal competitive grant awarded in 2020 (R.No. R/T/T/C/E/C/D/03/2013). The funders had no role in study design, data collection and analysis, decision to publish, or preparation of the manuscript. |

*Give information separately for cases and controls in case-control studies and, if applicable, for exposed and unexposed groups in cohort and cross-sectional studies.

**Note:** An Explanation and Elaboration article discusses each checklist item and gives methodological background and published examples of transparent reporting. The STROBE checklist is best used in conjunction with this article (freely available on the Web sites of PLoS Medicine at http://www.plosmedicine.org/, Annals of Internal Medicine at http://www.annals.org/, and Epidemiology at http://www.epidem.com/). Information on the STROBE Initiative is available at www.strobe-statement.org.
