# Supplementary material for: Evaluation of the diagnostic performance of EpiTuub® Fecal Rotavirus Antigen Rapid Test Kit in Amhara National Regional State, Ethiopia: A multi-center cross-sectional study
Source: PLoS One. 2023 Nov 30;18(11):e0295170. doi: 10.1371/journal.pone.0295170 (PMC10688889; doi:10.1371/journal.pone.0295170)
Supplement: Attachment — Submitted filename: Response to Reviewers.docx [file pone.0295170.s002.docx]

**To Editor of PLOS ONE**

Dear Sir/Madam,

The manuscript entitled “Evaluation of the diagnostic performance of EpiTuub® Fecal Rotavirus Antigen Rapid Test Kit in Amhara National Regional State, Ethiopia: A multi-center cross-sectional study**”** is an original research article authored by me and my colleagues were peer reviewed and review comments were received. On behalf of the authors, I would like to thank the Editor and the reviewers of the manuscript for their relevant and constructive comments. We hereby considered the comments from the editor and the reviewers. Please find our point-by-point responses to the comments.

Sincerely,

Debasu Damtie

**Response to the editor and the reviewers**

First of all, we would like to thank the editor and the reviewers of our manuscript for the invaluable and highly relevant comments which helped us to improve our manuscript. Below is our point-by-point response to the editor’s and the reviewer’s comments:

**Response to the editor:**

1. Please ensure that your manuscript meets PLOS ONE's style requirements, including those for file naming.

- **RESPONSE:** Thank you dear editor. We have considered the comment and edited the file naming styles other journal styles as per the template provided in the link.

2. We suggest you thoroughly copyedit your manuscript for language usage, spelling, and grammar. If you do not know anyone who can help you do this, you may wish to consider employing a professional scientific editing service.

- **RESPONSE:** Thank you dear editor. We have made a language edition by **Dr. Anastasia N. Vlasova (Associate Professor at The Ohio State University)**, one of the most senior members of the authors as per your recommendation.

**Response to the reviewers:**

**Reviewer #1**

1. The comparison between the Rotavirus A antigen using EpiTuub® 107 Fecal Rotavirus Antigen Rapid Test Kit (KTR-917, Epitope Diagnostics, Sandiago USA) and One-step RT-PCR is good, as the sensitivity rate reaches 98%, But in order to get a real comparison, this study must be compared with other available kits. In addition to, Some language mistakes and grammar mistakes I advise the revising for English Language by a native English speaker.

- **RESPONSE:** Thank you dear reviewer, as you mentioned it would have been better if we had evaluated as many kits as possible to identify the best performing kit among many. However, the scope of our study was to evaluate the EpiTuub® 107 Fecal Rotavirus Antigen Rapid Test Kit (KTR-917, Epitope Diagnostics, Sandiago USA) against one-step RT-PCR due to limited resources we have. Hence, we have indicated it as a limitation of this study and recommended further study involving as many kits as possible. As for the language edition, we have made language edition by one of the most senior members of the research team in the revised version of the MS.

2. Why the authors did not mentioned the catalogue number of QIAamp Mini spin viral RNA extraction kit and One step RT-PCR kit?

- **RESPONSE:** Dear reviewer, thank you for the comment. The Catalogue number of the QIAmp mini spin viral RNA extraction kit was (Qiagen, Hilden, Germany, Cat # 61904) and One step RT-PCR kit ((Bio-Rad,  [Hercules, California](http://en.wikipedia.org/wiki/Hercules,_California), United States‎, Cat # 1725151). We have indicated the catalogue number of the kits in the methods section of the revised version of the manuscript.

3. What the type of virus which used as positive control in the different method?

- **RESPONSE:** We have used rotavirus RNA extracted from known rotavirus A positive stool sample which was deposited at -80 ^o^c in Immunology and Molecular Biology Laboratory of the University of Gondar as a positive control for the one-step RT-PCR.

**Reviewer #2**

a. Causes of diarrheal diseases can be polymicrobial and the diagnosis of rotavirus does not exclude other viral or bacterial causes. The focus of the study was on rotavirus diagnosis using EpiTuub® Fecal Rotavirus Antigen Rapid Test Kit. I suggest the authors play down on statements that implies that rotavirus diagnosis excludes bacterial causes of diarrhea.

- **RESPONSE:** Dear reviewer, thank you for the comment. It is true that the cause of diarrhea is polymicrobial. Hence, investigation should involve all possible causes of diarrhea including viral causes. The current practice in Ethiopia is however focused only on investigation of parasitic and bacterial causes of diarrhea ignoring the viral causes of diarrhea. Of the viral causes, rotavirus is the leading cause of diarrhea and diagnosing rotavirus in addition to routine parasitic and bacterial investigations could help reduce empirical treatment of diarrhea in the study settings. Which in turn reduces the risk of antimicrobial resistance. Otherwise, we did not conclude that rotavirus diagnosis by itself rule out bacterial causes of diarrhea.

b. Table 1 mentioned the immunization status of the participants with regards to the Rotavirus RT-PCR results. The status of the participants as regards the rotavirus antigen test kit findings was not mentioned in the results or subsequent discussion. Hence, there is no basis from this study to conclude that the kit could be used in the evaluation and monitoring of rotavirus vaccine effectiveness

- **RESPONSE፡** Thank you dear reviewer for the comment. We have considered your comment and included the results for the rapid fecal rotavirus antigen test kit on Table 1. Of course, our focus was to evaluate the performance of the rapid rotavirus antigen test kit using one step RT-PCR as a gold standard. Accordingly, we have presented the test performance characteristics of the rapid test kit in the results section and discussed the findings. The test performance characteristics of the rapid antigen test kit in comparison to the gold standard was found to be acceptable. Hence, the test kit can be used to diagnose rotavirus infection among children. The WHO recommends use of a “case test-negative control” epidemiological study design for vaccine effectiveness studies in resource limited settings. Hence, our conclusion on the use of the kit for vaccine effectiveness studies is based on the aforementioned WHO’s recommendation in which the kit can be used to screen rotavirus positive cases and rotavirus negative controls (test negative controls) and trace back their immunization status retrospectively to determine rotavirus vaccine effectiveness in resource limited settings.

c. Concerning Question 2, The authors should elaborate on how their sample size of 537 was derived

- **RESPONSE:** Considering the single population proportion formula,

n = (Zα/_2_)^2^ P(1-P)

d^2^

Where n = sample size required; Zα/2 = standard normal variate for level of confidence (95%, 1.96), P = proportion of rotavirus among under five children with acute gastroenteritis (25%) (according to a previous study from reference 34); and d = margin of error (4%). We have also considered 10% non-response rate to get the final minimum sample size required for the study. Based on this formula, the minimum sample size calculated was 495. However, the hospitals involved in the study extended a little bit and collected 537 samples for our study. Hence, we considered all the 537 study participants to maximize the precision of the estimate.

d. Ethical consideration: What was the nature of the written communication from the University of Gondar research and publication office to the three referral hospitals

- **RESPONSE:** The letter written from the University of Gondar research and publication office explains the objectives and the relevance of the study and requests the hospitals for permission and cooperation in the study. We have indicated the nature of the letter in the revised version of the manuscript.
