# Supplementary material for: Evaluation of the diagnostic performance of EpiTuub® Fecal Rotavirus Antigen Rapid Test Kit in Amhara National Regional State, Ethiopia: A multi-center cross-sectional study
Source: PLoS One. 2023 Nov 30;18(11):e0295170. doi: 10.1371/journal.pone.0295170 (PMC10688889; doi:10.1371/journal.pone.0295170)
Supplement: Attachment — Submitted filename: Response to reviewers.docx [file pone.0295170.s003.docx]

**Response letter to the editor and reviewers**

PONE-D-23-19314R1

Evaluation of the diagnostic performance of EpiTuub® Fecal Rotavirus Antigen Rapid Test Kit in Amhara National Regional State, Ethiopia: A multi-center cross-sectional study

We would like to thank the editor and the reviewers of our manuscript for the invaluable and highly relevant comments which helped us to improve our manuscript. Below is our point-by-point response to the editor’s and the reviewer’s comments:

==============================
ACADEMIC EDITOR:

Line 25: antibiotics are not used to treat viral infections

**Response:** Thank you, dear Editor, we rewrite the sentence as per your comment.

Lines 87-89: Revise it to read University of Gondar, Felege Hiwot and Debre Markos Comprehensive Specialized Referral Hospitals

**Response:** thank you dear Editor, we have considered your comment and modified the manuscript accordingly.

Lines 89-90: indicate in detail how the sample size was arrived at

**Response:** Thank you, dear Editor, we have included how we determined the sample size in the revised manuscript.

Line 94-100: Any inclusion and exclusion criteria?

**Response:** Thank you, dear Editor, we have included the inclusion and exclusion criteria in the revised manuscript.

Line 95: indicate that for solid or formed stool 2g of stool was collected while in diarrheic or watery stool, 2ml of stool sample was collected

**Response:** Thank you, dear Editor, we have considered your suggestions in the revised manuscript.

Line 96: was the cryovial not overfilled? How did you placed a 2ml watery stool in a 2ml vial?

**Response:** We have collected approximately 2ml watery stool in a stool cup and transferred it into a 2 ml cryovial for storage. However, when we transfer the samples for storage, we added slightly lower volume than two ml to avoid overfilling of the cryovials.

Line 98: change ‘analysis’ to ‘analyses’

**Response:** We have accepted the change and considered it in the revised manuscript.

Line 99: change ‘assay’ to ‘assays’

**Response:** We have accepted the change and considered it in the revised manuscript.

Line 105/109: how did you homogenized the samples? Or you mean the samples were uniformly suspended?

**Response:** Dear Editor, it is just to mean samples were uniformly suspended. We have modified the statement to avoid any confusion.

Lines 105-111: The EpiTuub® Fecal Rotavirus Antigen Rapid Test Kit looks novel in your settings. Therefore, it is important to use schematic diagrams to describe how the test was performed. Visit this site to see example [Strep A Rapid Test Cassette (Control Line in Blue) JusChek Infectious Disease Rapid Test Kuala Lumpur (KL), Malaysia, Selangor Supplier, Suppliers, Supply, Supplies | Setia Scientific Solution](https://www.setiascientific.com/index.php?ws=showproducts&products_id=2841877&cat=Infectious-Disease-Rapid-Test&subcat=JusChek)

**Response:** Dear Editor, we have adapted the testing procedure from the kit insert leaflet and included schematic diagram to show the testing procedure of the kit.

Lines 131-133: A specific amplification with cycle threshold (CT) value of less than 40 was considered positive for rotavirus A infection. Provide reference for this statement.

**Response:** Thank you, dear Editor, we have cited references.

Line 144: Bring ‘Data entry and analysis’ before ‘Ethical considerations’

**Response:** Dear Editor, we have moved data entry and analysis section before ethical considerations section as per your considerations.

Tables 1-3: Headings look too long. Summarize

**Response:** We have modified and shortened the headings of the tables
